# Contractile effects and receptor analysis of adenosine-receptors in human detrusor muscle from stable and neuropathic bladders

**DOI:** 10.1007/s00210-016-1255-1

**Published:** 2016-05-17

**Authors:** Mahreen Pakzad, Youko Ikeda, Carly McCarthy, Darryl G. Kitney, Rita I. Jabr, Christopher H. Fry

**Affiliations:** Departments of Urology and Surgical Sciences, University College London, London, UK; School of Physiology, Pharmacology and Neuroscience, University of Bristol, Bristol, UK; Department of Biochemistry & Physiology, University of Surrey, Surrey, UK

**Keywords:** Adenosine, neuropathic bladder, detrusor, contraction, adenosine receptor

## Abstract

To measure the relative transcription of adenosine receptor subtypes and the contractile effects of adenosine and selective receptor-subtype ligands on detrusor smooth muscle from patients with neuropathic overactive (NDO) and stable bladders and also from guinea-pigs. Contractile function was measured at 37°C in vitro from detrusor smooth muscle strips. Contractions were elicited by superfusate agonists or by electrical field stimulation. Adenosine-receptor (A1, A2A, A2B, A3) transcription was measured by RT-PCR. Adenosine attenuated nerve-mediated responses with equivalent efficacy in human and guinea-pig tissue (pIC50 3.65–3.86); the action was more effective at low (1–8 Hz) compared to high (20–40 Hz) stimulation frequencies in human NDO and guinea-pig tissue. With guinea-pig detrusor the action of adenosine was mirrored by the A1/A2-agonist N-ethylcarboxamidoadenosine (NECA), partly abolished in turn by the A2B-selectve antagonist alloxazine, as well as the A1-selective agonist N6- cyclopentyladenosine (CPA). With detrusor from stable human bladders the effects of NECA and CPA were much smaller than that of adenosine. Adenosine also attenuated carbachol contractures, but mirrored by NECA (in turn blocked by alloxazine) only in guinea-pig tissue. Adenosine receptor subtype transcription was measured in human detrusor and was similar in both groups, except reduced A2A levels in overactive bladder. Suppression of the carbachol contracture in human detrusor is independent of A-receptor activation, in contrast to an A2B-dependent action with guinea-pig tissue. Adenosine also reduced nerve-mediated contractions, by an A1- dependent action suppressing ATP neurotransmitter action.

**Electronic supplementary material:**

The online version of this article (doi:10.1007/s00210-016-1255-1) contains supplementary material, which is available to authorized users.

## Introduction

The purinergic system regulates bladder function through the action of ATP or its metabolites. ATP and its immediate breakdown product ADP have been extensively studied through their respective actions on purinergic P2X and P2Y receptors. ATP is a functional excitatory neurotransmitter in most animal and also human overactive bladders (Bayliss et al. 1999; Fry et al., 2011). ATP is also released from urothelium in response to mechanical and chemical interventions (Ferguson et al. 1997; Sadananda et al. 2009) and this is greater from tissues obtained from pathologies such as overactive bladder (Munoz et al. 2011). ADP has a negative inotropic effect on detrusor smooth muscle (McMurray et al., 1998) but activates suburothelial interstitial cells and also increases spontaneous bladder contractions (Fry et al. 2012).

Adenosine, a breakdown product of ATP via endonucleotidases, is a P1 receptor agonist. It relaxes agonist-induced detrusor contractions (Rubinstein et al. 1998) and attenuates stretch-activated urothelial ATP release (Dunning-Davies et al. 2013). Endonucleotidase activity is reduced in human detrusor from overactive bladders (Harvey et al. 2002) and variation of adenosine levels may have significant consequences on detrusor contractile function. However, its action on human detrusor from normal or overactive bladders has not been thoroughly investigated. The P1 class of adenosine receptors (A1, A2_A_, A2_B_ and A3) mediate their actions via different G-protein-coupled intracellular pathways (Ralevic and Burnstock 1998). This study described the contractile effects of adenosine, via these receptor subtypes, in isolated human detrusor from patients with stable bladders and those with neuropathic detrusor overactivity. In addition, the expression of P1-receptor subtypes in these samples was measured. Functional experiments with well-characterised guinea-pig detrusor were also done to: establish optimal concentrations of adenosine receptor ligands; and assist with the interpretation of human detrusor data.

## Methods

### Tissue samples and ethical approvals

Human bladder tissue was collected with informed and signed patient consent. All procedures were in accordance with ethical committee approval of University College London Hospitals, and with the 1964 Helsinki declaration. Biopsies were obtained from the bladder dome of patients who had: i) bladder carcinoma undergoing cystectomy and symptomatically-stable bladders; the biopsy was distant from the tumour (*n* = 16: 9 female; 7 male; age 56 ± 14 yr); ii) spinal cord injury or multiple sclerosis undergoing clam ileocystoplasty with urodynamically-demonstrated neuropathic detrusor overactivity (NDO; *n* = 18: 8 female; 10 male; age 33 ± 7 yr). NDO patients were significantly (*p* < 0.05) younger than stable bladder patients. Samples were immediately stored in Ca^2+^-free HEPES-Tyrode’s solution, the mucosa removed and portions either frozen in liquid N_2_ or used for functional experiments begun generally 30–60 min after biopsy retrieval, and never more than two hours. Animal experiments used guinea-pig (Dunkin-Hartley, males, 350–450 g) bladders removed after euthanisation by cervical dislocation in accordance with UK Home Office procedures (UK Animals (Scientific Procedures) Act, 1986). Animals were housed two to three per cage with *ad libidum* access to water and standard chow in a pathogen-free unit of 12/12 h light-dark cycle.

### Measurement of contractile function

Detrusor strips (4–5 mm length, ≤0.5 mm diam.) were tied to an isometric force transducer and superfused with Tyrode’s solution at 36°C. Nerve-mediated contractions (TTX-sensitive) were generated by electrical field stimulation (0.1 ms pulses, 1–40 Hz, 3-s train every 90-s). Agonist-induced responses were generated by addition of the muscarinic receptor agonist, carbachol. Nerve-mediated contraction magnitude, *T*, as a function of frequency, *f*, was fitted to: , where *T*_max_ is maximum tension at high frequencies and *f*_1/2_ the frequency to attain *T*_max_/2, *n* is a constant. Ratios of tension at low (4 Hz) and near maximum (40 Hz) frequencies (T_4_/_40_) for human and 4 Hz and 20 Hz (T_4_/_20_) for guinea-pig detrusor were also calculated. T_4_/_20_ was chosen for guinea-pig as *T*_40_ contractions can have a significant TTX-insensitive component, indicative of direct muscle stimulation, and *T*_20_ contractions are near maximal.

### Solutions and reagents

Tyrode’s solution contained (mM): NaCl, 118; KCl, 4.0; NaHCO_3_, 24; NaH_2_PO_4_, 0.4; MgCl_2_, 1.0; CaCl_2_, 1.8; glucose, 6.1; Na pyruvate, 5.0; 95%O_2_,5%CO_2_; pH 7.40 ± 0.02. HEPES-Tyrode’s was similar except NaHCO_3_ was replaced with HEPES (10 mM) plus NaCl (14 mM), titrated to pH 7.4 with NaOH and gassed with 100% O_2_. Stock solutions (1 mM in DMSO) of the selective A-receptor ligands N-ethylcarboxamidoadenosine (NECA, A1/A2-selective agonist); N6-cyclopentyladenosine (CPA, A1-selective agonist); 1,3-dipropyl-8-cyclopentylxanthine (DPCPX, A1-selective antagonist); CGS 21680 (A2_A_–selective agonist); ZM-241,385 (A2_A_–selective antagonist); alloxazine (A2_B_ antagonist); IB-MECA (A3–selective agonist): and stock solutions (1–10 mM in Analar grade water) of atropine, carbachol, adenosine and ABMA were diluted in Tyrode’s solution. Test concentrations for adenosine and NECA were at the higher range of the dose-response curve (see Results) or similar to those derived from the literature. All reagents were from Sigma-Aldrich (UK).

### Quantification of adenosine receptor expression

This was done by RT-PCR and Western blotting. Details of the methodology and the primers and antibodies used are included in the supplementary material.

### Data and statistics

Data are mean ± SD, differences between data sets were examined with paired or unpaired Student’s *t*-tests; the null hypothesis was rejected at *p* < 0.05. *n*-values refer to the number of preparations, one each from separate patients or animals.

## Results

### *Baseline data*: *nerve-mediated contractions*

**Fig 1:**
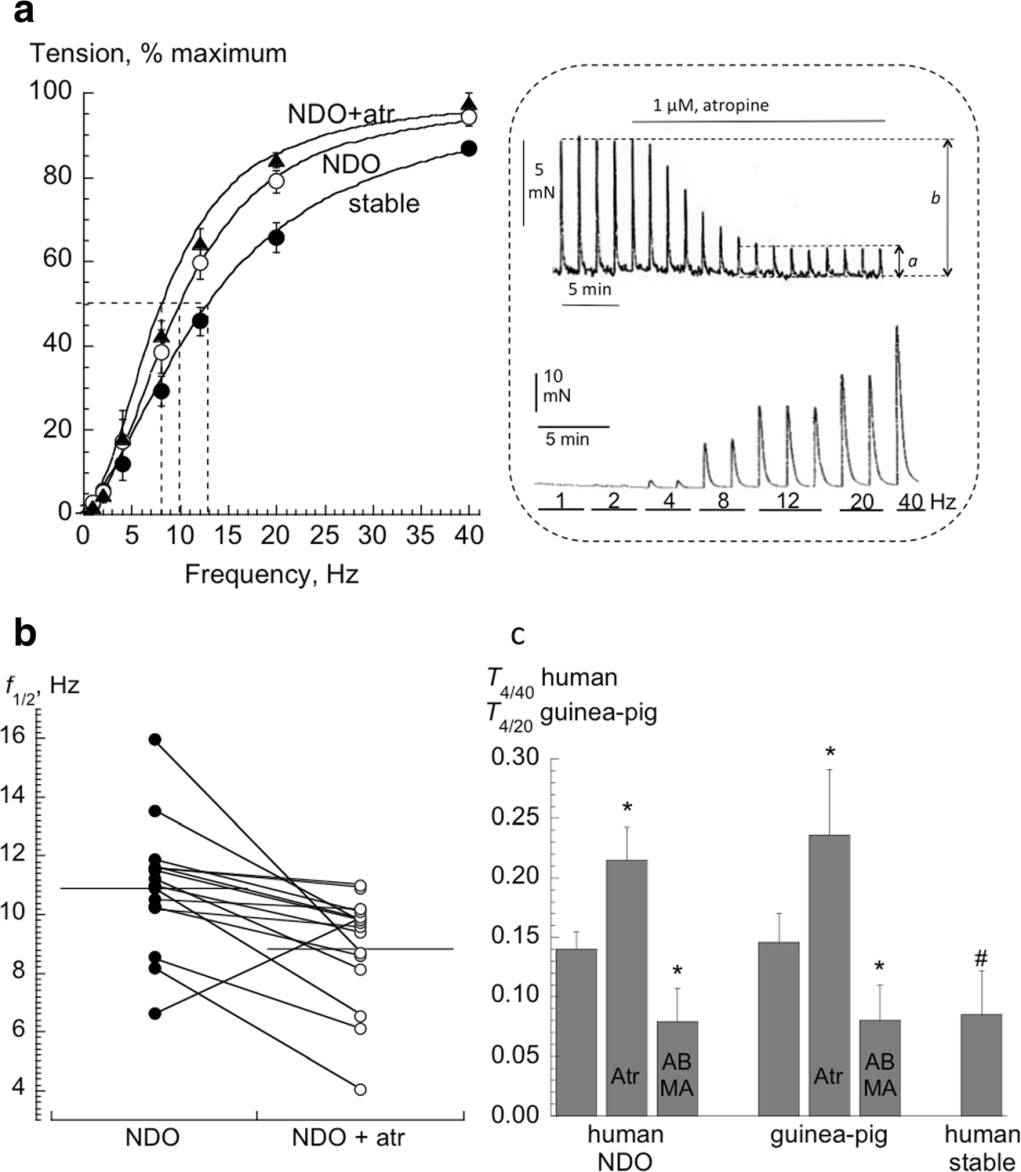
Force-frequency relations of human detrusor from stable and NDO bladders. **a:** force-frequency curves of human detrusor from stable (*n* = 16) or NDO (*n* = 18) bladders, and detrusor from NDO bladders treated with atropine (atr, *n* = 17). Inset: upper panel, nerve-mediated contractions (12 Hz stimulation) from an NDO bladder preparation, atropine was added as shown. The ratio of a*100/*b* is the atropine resistant value. Lower panel, a force-frequency response with two of three contractions at each frequency, except a single one at 40 Hz. **b:** *f*_1/2_ values for detrusor from NDO bladders in the absence (NDO) and presence of atropine (NDO atr, *n* = 17). Lines connect values from the same preparations. **c:** Values (*n* = 17) of the ratio *T*_4/40_ for human and *T*_4/20_ for guinea-pig detrusor. Human NDO and guinea-pig values are also shown in the presence of atropine (atr) or ABMA. Values from stable bladder preparations (*n* = 16) are also shown; *p < 0.05 vs values in the absence of atropine or ABMA: #*p* < 0.05 human stable vs NDO detrusor in the absence of atropine or ABMA

Table 1 shows nerve-mediated contraction data and adenosine potency to reduce contractions. Maximum tension was similar from human stable and NDO, and also in guinea-pig detrusor. The frequency for half-maximum tension, *f*_1/2_, was significantly greater with detrusor from stable human bladders compared to human NDO and guinea-pig detrusor: *f*_1/2_, values from the latter groups were not significantly different. Atropine-resistant contractions in human NDO (Fig 1a inset, upper panel) and guinea-pig detrusor were abolished by P2X_1_ receptor desensitisation with 1 μM ABMA - consistent with ATP as a functional neurotransmitter. Fig 1a shows force-frequency plots for detrusor from human stable and NDO (sample in inset, lower panel) bladders; the curve for NDO detrusor was left-shifted and moreover shifted further to the left in the presence of atropine, with a reduction of *f*_1/2_ recorded in 16 of 17 NDO preparations (Fig 1b). A similar reduction of *f*_1/2_ by atropine, from 9.2 ± 3.9 to 6.0 ± 2.5 Hz (*n* = 24), was seen in guinea-pig tissue.

**Table 1:** Baseline contraction characteristics. Nerve-mediated contractions and potency of adenosine to reduce contractions in detrusor smooth muscle from human and guinea-pig bladders. NDO: neuropathic detrusor overactivity. Mean data ± SD; *p < 0.05, **p < 0.01 vs NDO human bladder. Values in parenthesis are the number of preparations

	Human stable bladder	Human NDO bladder	Guinea-pig bladder
*T*_max_, mN.mm^−2^	34.6 ± 21.1 (16)	44.3 ± 22.6 (18)	42.1 ± 13.8 (24)
*f*_1/2_, Hz	13.7 ± 4.3 (16)	10.9 ± 2.1 (18) *	9.2 ± 3.9 (24) **
Atropine resistance, %	2.4 ± 1.9 (16)	37.4 ± 22.5 (17) **	53.8 ± 15.4 (9) **
*T*_4/40_	0.102 ± 0.015 (16)	0.140 ± 0.015 (18) **	0.167 ± 0.062 (24) **
Adenosine pIC_50_	3.70 ± 0.52 (5) (IC_50_ = 199 μM)	3.65 ± 0.54 (5) (IC_50_ = 224 μM)	3.86 ± 0.33 (11) (IC_50_ = 138 μM)

The ratio of tension (*T*) at 4 and 40 Hz stimulation (*T*_4/40_) quantified low and high frequency responses whereby larger values mean proportionately more tension at 4 Hz compared to 40 Hz stimulation (Table 1). With human NDO tissue *T*_4/40_ was significantly increased by atropine and reduced by ABMA (Fig 1c). Similar observations were made for guinea-pig tissue: *T*_4/20_ in control was 0.128 ± 0.0448 and increased by atropine (0.236 ± 0.109, *n* = 9, *p* < 0.01) or decreased by ABMA (0.080 ± 0.030, *n* = 9, *p* = 0.005). With detrusor from stable human bladders the *T*_4/40_ ratio was smaller than that of NDO and guinea-pig tissues, but similar to the NDO + ABMA value (Fig 1c), was unaffected by ABMA and could not be determined in the presence of atropine, due to the lack of atropine-resistant contractions. These data are consistent with ATP neurotransmitter release being proportionately greater compared to acetylcholine at lower stimulation frequencies.

### Contractile effects of adenosine

**Fig 2:**
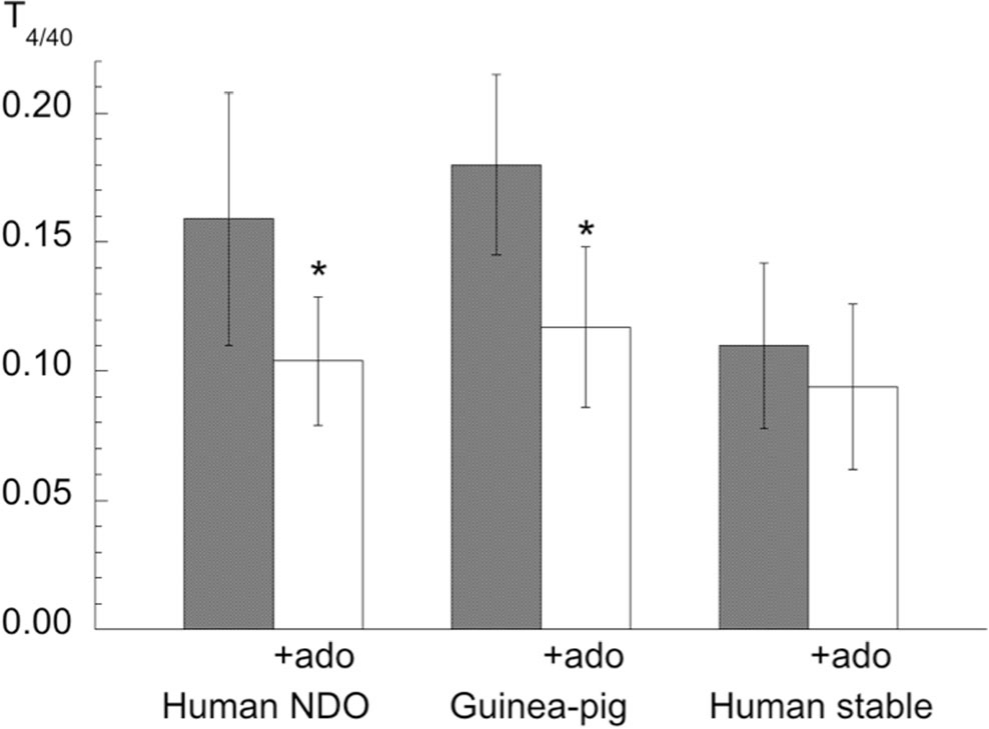
Frequency-dependent effects of adenosine on nerve-mediated contractions. *T*_4/40_ values (T_4_/T_20_ for guinea-pig detrusor) in the absence and presence of adenosine (ado). Data from human NDO, guinea-pig and human stable bladder preparations. *p < 0.05 in the absence of adenosine

Adenosine reduced the magnitude of nerve-mediated contractions in a dose-dependent manner (Table 1), with pIC_50_ values similar in human and guinea-pig tissues; a concentration of 1 mM was used in subsequent experiments. A pIC_50_ value (6.27 ± 0.49, *n* = 10) for the A1/A2 receptor agonist NECA was also generated with guinea-pig detrusor; a test value of 10 μM was subsequently used. Adenosine exerted a frequency-dependent effect on tension reduction in human NDO (*n* = 10) and guinea-pig (*n* = 12) detrusor, where acetylcholine (ACh) and ATP are both neurotransmitters (Fig 2). There was a greater action at low frequencies, so that *T*_4/40_ ratios (T_4_/T_20_ for guinea-pig deturosr) were significantly (*p* < 0.05) reduced. By contrast there was no effect on the *T*_4/40_ ratio with human stable detrusor (*n* = 15) where only ACh is a neurotransmitter. These data are consistent with the hypothesis that an action of adenosine in guinea pig and NDO human bladders is to reduce preferentially ATP, rather than ACh, release from the motor nerve terminal. The action of adenosine via A-receptor subtypes was further characterised using three modes of contractile activation: direct detrusor smooth muscle activation by carbachol (0.3 μM); *T*_40_ contractions for those mediated largely by ACh; *T*_4_ contractions for those with a greater purinergic component.

### *Adenosine and A-receptor subtype-selective agents*: *contractions from human stable bladder detrusor*

**Fig 3:**
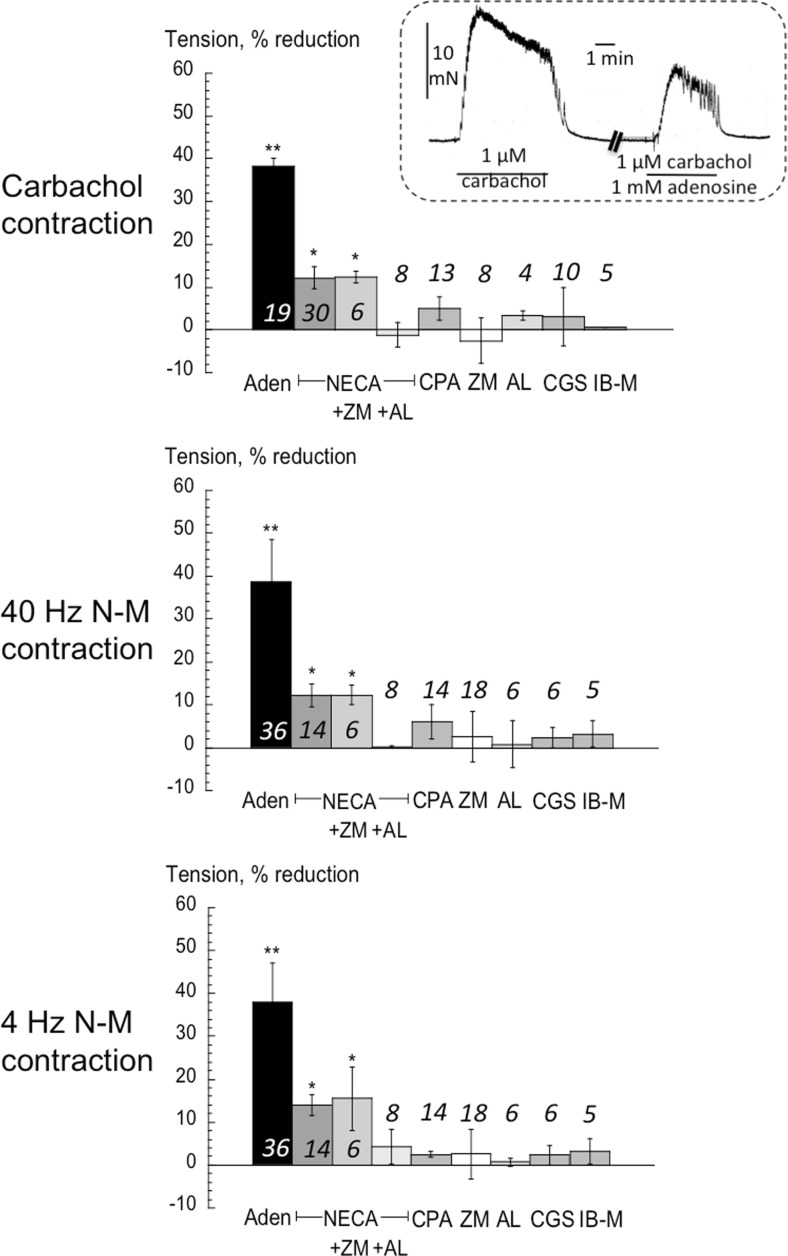
The effect of adenosine and selective A-receptor subtype ligands on detrusor from human stable bladders. Values are reduction of tension from control. Three sets of data refer to actions on the carbachol contracture (top panel); the *T*_40_ NM-C centre panel) and *T*_4_ Hz NM-C (bottom panel). Mean data ± SD, *p < 0.05 vs control, **p < 0.01 vs control. Values above bars are number of preparations. ZM = ZM241385; AL = alloxazine; CGS = CGS-21,680; IB-M = IB-MECA

Fig 3 shows data from responses from stable human bladder detrusor. Adenosine reduced carbachol contractures (top panel and inset) by about 40%, but this was only partially matched by the A1/A2-receptor agonist NECA. The effect of NECA was unaffected by the A2_A_ antagonist ZM-241,385 (ZM; 0.1 μM), but abolished completely by the A2_B_ antagonist alloxazine (AL; 1 μM). The A1-selective agonist (CPA, 10 μM) was without significant effect. The actions of these agents were mirrored with *T*_40_ and *T*_4_ contractions (Fig 3, centre and bottom panels), consistent with the fact that in human stable bladders nerve-mediated contractions are entirely cholinergic. ZM-241,385 and alloxazine alone were without effect (data not shown). The selective agonists to A2_A_ (CGS-21,680, 10 μM) and A3 (IB-MECA, 10 μM) receptors were also without significant effects on any contractile variable.

### *Adenosine and A-receptor subtype-selective agents*: *contractions from human NDO detrusor*

**Fig 4:**
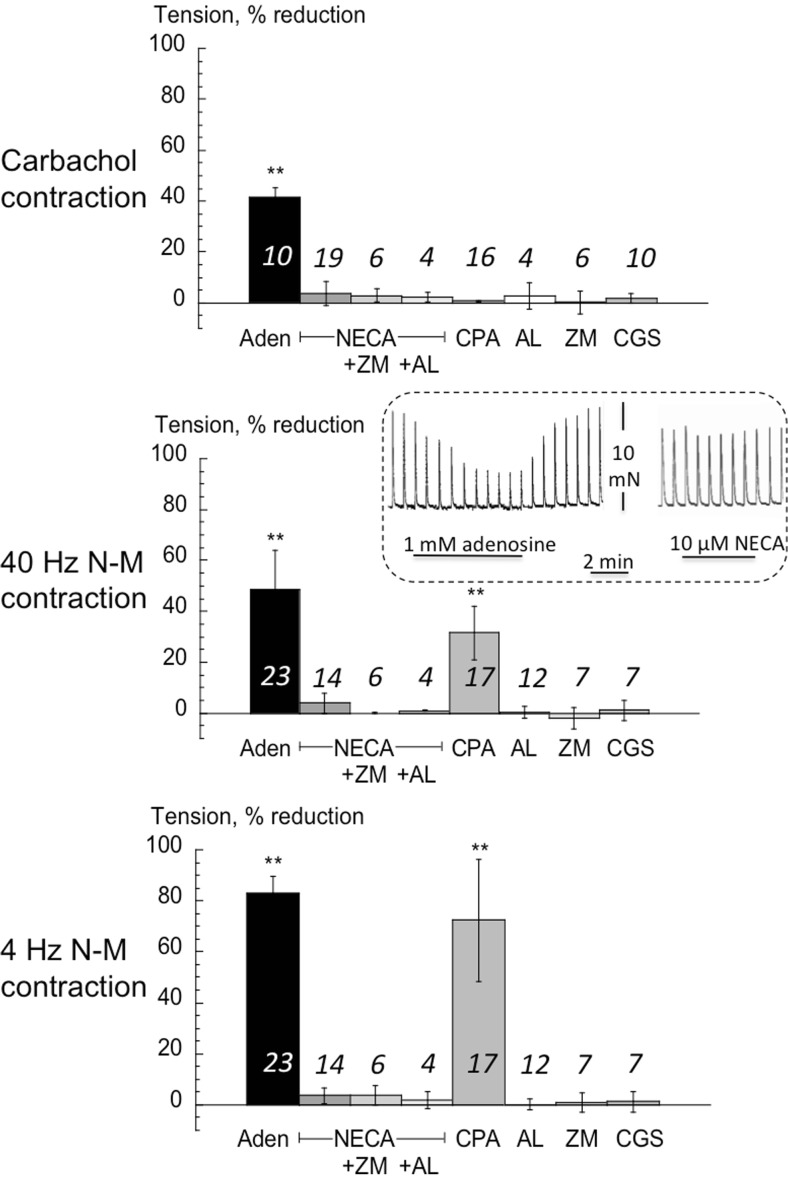
The effect of adenosine and selective A-receptor subtype ligands on detrusor from human NDO bladders. Values are reduction of tension from control. Three sets of data refer to actions on the carbachol contracture (left panel); the *T*_40_ NM-C centre panel) and the *T*_4_ NM-C (right panel). Mean data ± SD, **p < 0.01 vs control. Values above bars are number of preparations. ZM = ZM241385; AL = alloxazine; CGS = CGS-21,680; IB-M = IB-MECA

The pattern of responses (Fig 4) showed differences from those of stable bladder. Adenosine reduced carbachol contractions in NDO detrusor by a similar amount to detrusor from stable bladders, however NECA and CPA were both without any significant effect. Adenosine had a similar effect on *T*_40_ contractions (Fig 4 inset), however it had a much greater action on *T*_4_ contractions (*p* < 0.001; 4 vs 40 Hz). Furthermore, CPA also reduced nerve-mediated contractions: more on *T*_4_ compared to *T*_40_ contractions (*p* < 0.001; 4 vs 40 Hz); NECA again was without effect on *T*_40_ (inset) or *T*_4_ contractions. ZM-241,385, alloxazine, CGS-21,680 and IB-MECA were without effect.

### *Adenosine and A-receptor subtype-selective agents*: *contractions from guinea-pig detrusor*

**Fig 5:**
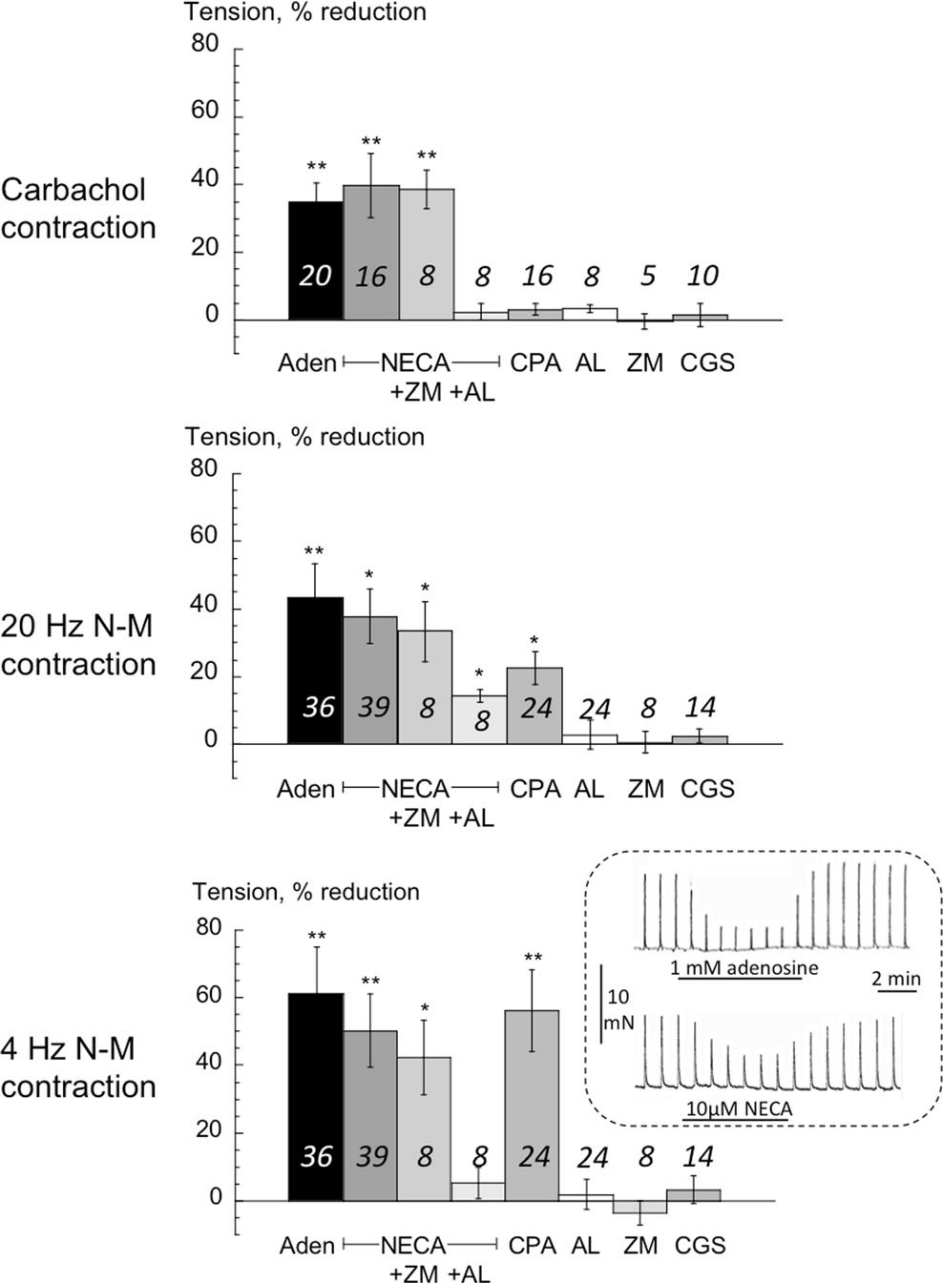
The effect of adenosine and selective A-receptor subtype ligands on detrusor from guinea-pig bladders. Values are reduction of tension from control. Three sets of data refer to actions on the carbachol contracture (left panel); the *T*_20_ NM-C centre panel) and the *T*_4_ NM-C (right panel). Mean data ± SD, *p < 0.05 vs control, **p < 0.01 vs control. Values above bars are number of preparations. ZM = ZM241385; AL = alloxazine; CGS = CGS-21,680; IB-M = IB-MECA

Fig 5 shows a similar protocol, except *T*_20_ and *T*_4_ contractions were analysed (see Methods). Reduction by adenosine of the carbachol contracture and *T*_20_ contractions were similar to those with human detrusor. However, in this tissue NECA was equally effective (Fig 5 inset), and its own action was unaffected by ZM-241,385. Alloxazine blocked completely the effect of NECA on the carbachol contracture and *T*_4_ contractions, and was partially effective on *T*_20_ contractions. CPA had no effect on the carbachol contracture, but did reduce *T*_20_ contractions and to a greater extent *T*_4_ contractions, similar to its action on human NDO detrusor. ZM-241,385, alloxazine, CGS-21,680 and IB-MECA alone were without effect on any contraction. Control experiments showed that the selective A1 receptor antagonist, DPCPX antagonised the effects of CPA and NECA on nerve-mediated contractions. Schild plots yielded pA_2_ values of 9.62 and 8.54 respectively for the action of DPCPX, i.e. the K_b_ values were 0.24 and 2.9 nM, respectively, similar to those previously reported (Nicholls et al., 1996).

### Adenosine receptor gene transcription in stable and NDO human bladder samples

**Fig 6:**
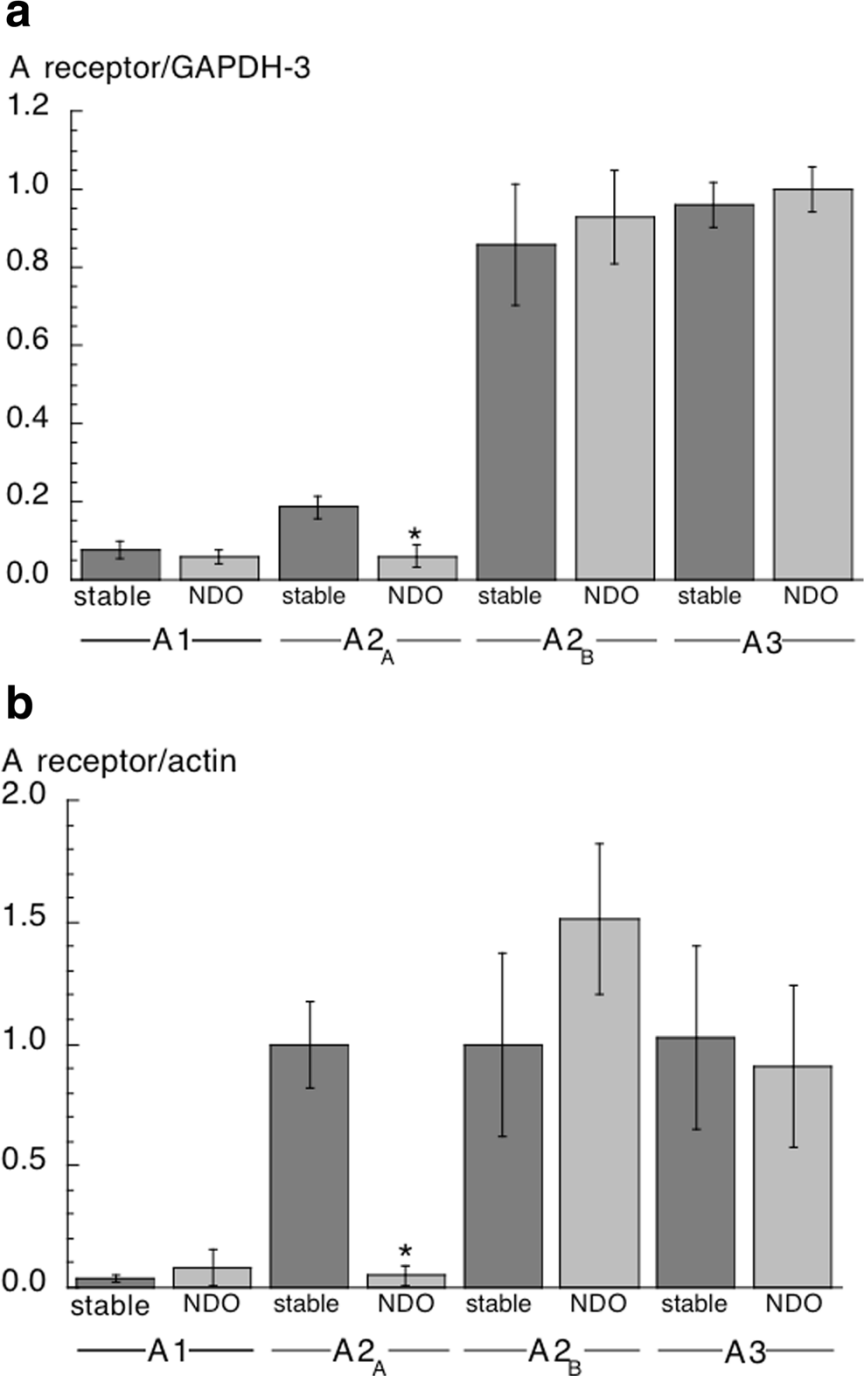
Amplified gene products and protein levels for A1, A2_A_, A2_B_ and A3 receptors in human detrusor. **a:** RT-PCR data of adenosine (A) receptor subtype expression in samples from human stable and NDO bladders. Data are shown as a ratio of GAPDH-3. **b:** A-receptor subtype protein levels in samples from human stable and NDO bladders. Data are shown as a ratio of actin product. *p < 0.05 stable vs NDO; (*n* = 6,7 for both parts)

Stable and NDO samples yielded similar quantities of RNA (1.40 ± 0.16 vs 1.73 ± 0.60 μg.mg^−1^ respectively, *n* = 6,7). RNA from each sample was run on two separate gels and the average value used. Amplified A-receptor gene products, normalised to their own GAPDH-3 band, showed that the A1 receptor was expressed the least and A2_B_/A3 the most (Fig 6a). All receptor subtypes were equally transcribed in stable and NDO groups, except for A2_A_ that was significantly reduced in NDO samples. Adenosine receptor protein expression was measured in the same samples (*n* = 6,7). As with gene transcription data, protein expression of the A1-receptor was very low but with no difference between the two groups. There was greater expression of the other receptors and, as above, the A2_A_ level was significantly reduced in the NDO group, but A2_B_ and A3 levels were similar in the two groups (Fig 6b).

## Discussion

### Frequency-dependence of transmitter release

Nerve-mediated detrusor contractions from stable human bladders were mediated mainly by ACh, but human NDO and guinea-pig detrusor showed an additional ATP-dependent component (Bayliss et al. 1999; Fry et al., 2011). ATP-mediated contractions were more prominent at low stimulation frequencies, suggesting a frequency-dependent release of ATP and ACh, as also observed with ATP and noradrenaline release at sympathetic terminals (Westfall et al. 1996; Todorov et al. 1999; Stjärne 2001). This may underlie the ATP-dependence of the initial phase of nerve-mediated contractions in some detrusor preparations (Tsai et al. 2012).

### Adenosine and detrusor contractions

Adenosine reduced contractions in all detrusor preparations. Carbachol and *T*_40_ (T_20_ for guinea-pig detrusor) contractions, the latter dominated by ACh release, were equally reduced by about 40%, as were *T*_4_ contractions from stable bladders. This suggests a direct effect of adenosine on detrusor muscle (Nicholls et al. 1996; Woods et al. 2003). However, *T*_4_ contractions in human NDO and guinea-pig detrusor were reduced to a greater extent, consistent with the hypothesis that adenosine additionally reduced ATP release from motor nerves, similar to its action on sympathetic nerve terminals (Driessen et al. 1994).

### Adenosine receptor subtypes mediating contractile responses to carbachol

The A1/A2 agonist NECA reduced the carbachol contraction to the same extent as adenosine in guinea-pig detrusor. However, in human tissue it had a much smaller effect on stable bladder tissue and none at all in NDO detrusor. Any negative inotropic effect was abolished by the A2_B_ receptor antagonist alloxazine, but not by an A2_A_ receptor antagonist, ZM-241,385. An A2_A_ agonist (CGS-21,680) and an A3 agonist (IB-MECA) were without effect; A2_B_ agonists were not used due to their poor selectivity. These data suggest that A2_B_ receptors mediate the effect of adenosine in guinea-pig detrusor, as in other animal smooth muscles (Nicholls et al. 1996; Aronsson et al., 2010). However, with human detrusor from stable and NDO bladders A2_B_ receptors mediate only part of the effect of adenosine or not at all, respectively. It is possible that the selective adenosine receptor ligands were less potent, relative to that of adenosine, in human detrusor, especially from NDO bladders. However, higher concentrations were equally ineffective (not shown). These observations demonstrate the uncertainty in extrapolating data from animal tissue to explain contractile pathways in human detrusor.

### Adenosine receptor subtypes mediating nerve-mediated contractile responses

High-frequency nerve-mediated, *T*_40_, contractions from human stable bladder and NDO bladder preparations and low-frequency, *T*_4_, contractions from stable bladder are dominated by ACh release. Data were similar to those obtained by application of carbachol, thus no additional effect of adenosine on ACh release from motor nerves is proposed. However, *T*_4_ contractions from NDO human and guinea-pig detrusor had a significant ATP-dependent component and were reduced by adenosine to a greater extent than contractions dependent only on ACh.

NECA had a similarly enhanced effect in guinea-pig tissue, as did the more selective A1-receptor agonist CPA in both human NDO and guinea-pig tissue. A1 receptors are implied in regulation of transmitter release from parasympathetic nerves in human and murine detrusor, although a direct role on detrusor muscle cannot be excluded (Searl et al., 2016). These data are consistent with the hypothesis that adenosine and CPA reduced *T*_4_ contractions by limiting preferentially nerve-mediated release of ATP, rather than ACh. NECA had an equivalent effect on guinea-pig tissue, but its lack of effect on human NDO *T*_4_ contractions, corroborates its diminished effect on human detrusor ACh-mediated responses.

The RT-PCR and protein expression data show that variable transcription and expression of A-receptors are unlikely to underlie different responses to adenosine, and to NECA or CPA in human detrusor from stable and NDO bladders. Functional experiments showed that A2_B_ receptors mediated all or part of the contractile response to adenosine on guinea-pig and human stable detrusor, and A1 receptors mediated attenuation of neurotransmitter, predominantly ATP, release. However, expression and translation of A1, A2_B_, and also A3 receptors were equivalent in the two groups. Only the A2_A_ receptor was down-regulated in NDO tissue and this had no functional counterpart in contractile regulation.

**Fig 7:**
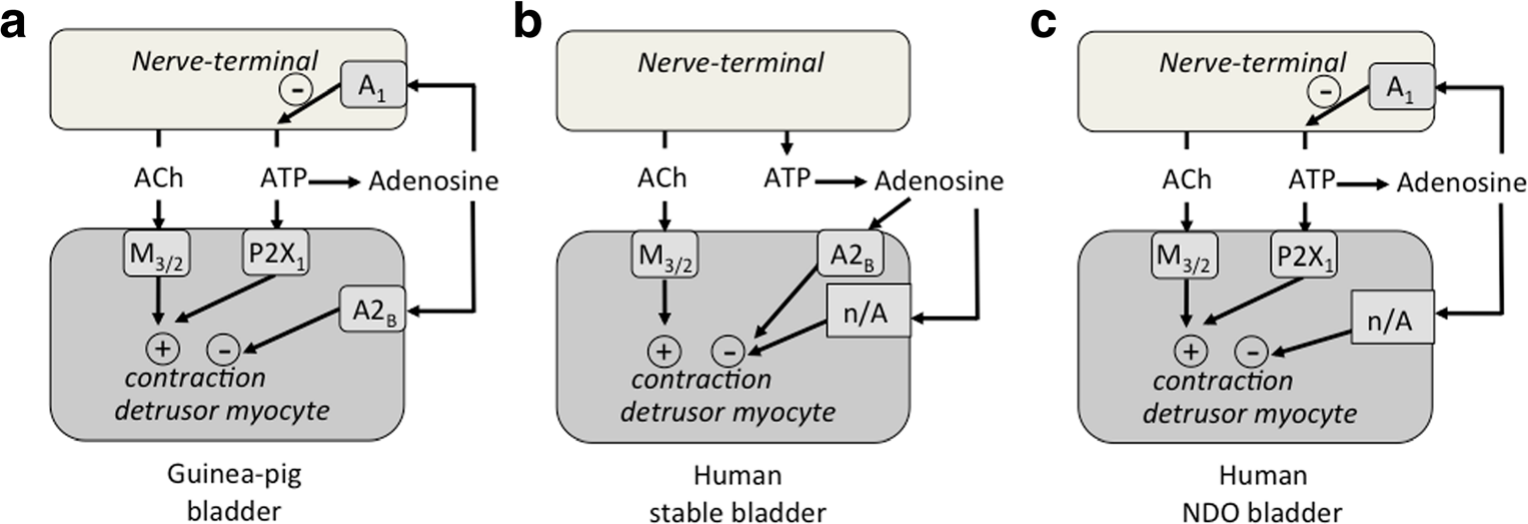
A model of the effects of adenosine on nerve-endings and smooth muscle to modulate contractile function in human and guinea-pig detrusor. **a:** Guinea-pig bladder. Acetylcholine (ACh) and ATP bind to M_3_ and P2X_1_ receptors respectively. Adenosine, derived from ATP breakdown in the nerve-muscle junction, binds to A2_B_ (detrusor) and A1 (nerve endings) receptors. **b:** Human stable bladder. ACh binds to M_3_ receptors; ATP is completely broken down in the nerve-muscle junction. Adenosine has an effect via non-A receptors (n/A) on detrusor. **c:** Human NDO bladder. ACh binds to M_3_ receptors; ATP is incompletely broken down and activates detrusor via P2X_1_ receptors. Adenosine acts on detrusor via non-A receptors and binds to nerve-terminal A1 receptors. + activation, − inhibition

Fig 7 summarises the actions of adenosine on human and guinea-pig detrusor contractile function. With guinea-pig detrusor (part a) motor nerve excitation releases acetylcholine and ATP, both of which exert functional effects on detrusor. In addition, adenosine as a metabolite of ATP relaxed detrusor via an A2_B_ receptor and, we propose, attenuated selectively release of ATP from the motor nerve, via an A1 receptor. With human detrusor from stable bladders (part b), acetylcholine is the sole functional transmitter, whereby it acts importantly via M3 receptors (Chess-Williams et al. 2001) but with a contribution from M2 receptors (Pontari et al., 2004). ATP is completely hydrolysed (Harvey et al. 2002) and adenosine could relax detrusor itself. However, in contrast to guinea-pig detrusor, only in a small part via an A2_B_ receptor, most of the effect was via a non-A receptor (n/A) pathway. With NDO bladder detrusor (part c) acetylcholine also acts via M3 receptors and with a larger contribution from M2 receptors (Pontari et al., 2004). In addition, not all ATP released from excitatory nerves is hydrolysed and some activates detrusor via a P2X_1_ receptor (King et al. 2004). Adenosine relaxed detrusor exclusively via an n/A pathway and also acted on the nerve terminal, via an A1 receptor, to attenuate exclusively ATP release. In principle, an A1 receptor modulator could be a drug target for the human overactive bladder (OAB). It would exert no direct effect on detrusor muscle, but would attenuate the release of ATP from nerve-terminals, the transmitter associated with OAB in humans. However, to propose an agent as an in vivo modulator of normal and pathological function requires a more integrated understanding of its actions. For example, A1 and A2_A_ receptor agonists modulate nervous control of the micturition reflex at spinal and supraspinal sites (Kitta et al. 2012; Kitta et al. 2014).

### Study limitations

A-receptor ligands were used at high concentrations with respect to their dose-range on the primary receptor to ensure a full effect, but they may also exert non-specific effects via other receptors. Some effects of actions of adenosine were independent of known A-receptor sub-types and the basis of this non-specific effect remains unknown. The limited size of the biopsy samples limited further investigation of the cell pathways and is the subject of further studies. The age of NDO patients was significantly less than that of stable bladder patients, as expected from the two cohorts of patients from whom samples were obtained. However, a previous study has recorded no age-dependence of basic contractile characteristics over the ranges recorded here (Fry et al. 2011). Interpretation of the data in terms of adenosine modulating transmitter release from nerve-terminals is indirect using tension as a primary experimental variable. Direct measurement of transmitter release from the very small available human biopsy samples will validate, or otherwise, the conclusions of this study when sensitive systems become validated.

## Electronic supplementary material

ESM 1 (DOCX 132 kb)
