# Supplementary material for: Contractile effects and receptor analysis of adenosine-receptors in human detrusor muscle from stable and neuropathic bladders
Source: Naunyn Schmiedebergs Arch Pharmacol. 2016 May 17;389:921–9. doi: 10.1007/s00210-016-1255-1 (PMC4939168; doi:10.1007/s00210-016-1255-1)
Supplement: Supplementary file 1 — (DOCX 132 kb) [file 210_2016_1255_MOESM1_ESM.docx]

*Quantification of adenosine receptors*. Frozen samples were weighed and crushed in liquid N_2_. Total RNA, DNA and protein fractions were isolated using a modified single-step liquid-phase separation method (Chomczynski and Sacchi 1987). Briefly, TRI Reagent (Sigma, UK) was added to the samples (1 ml per 50-100 mg tissue) and centrifuged for 10 min (4°C, 12,000g). Total RNA (aqueous phase) and protein (organic phase) amounts were quantified by RT-PCR and western blotting, respectively. Total RNA concentration and purity was measured by recording absorbance at 260/280 nm, only total RNA samples with a ratio of >2.0 was used. Protein quantity was measured with a Bradford protein micro-assay (Bio-Rad, UK).

*Reverse transcription-polymerase chain reaction (RT-PCR)*. RNA samples (4 μg/μl) were mixed with nuclease-free double-distilled H_2_O (22 µl) and primers (1 µl, Table 1) then heated with a blend of deoxyribonucleotide triphosphates (dNTP, 2 µl, 10 mM) to 70°C (10min) and subsequently at 42°C (2min). RT was initiated by adding 2 μl of the mix to a 2:1 solution of 5x M-MLV RT buffer and 0.1 M dithiothreitol (42°C, 50min) and terminated by heating to 75°C for 15min. The produced complementary DNA (cDNA) samples were then stored at -20°C. Amplification was performed at 94°C for 4min and 30 cycles at 95°, 60°C and 72°C each for 1 min in a medium containing (µl): cDNA, 2.0; forward primer, 0.5; reverse primer, 0.5; Taq polymerase, 0.5 in 10xPCR buffer, 4.5 and 25mM MgCl_2_, 4.0; 200 μM dNTP, 1.0; ddH_2_O, 36.5. Ileal mRNA was used as a positive control. Amplified products were separated by 1.5% agarose gel electrophoresis and stained with ethidium bromide. The integral optical density of the cDNA bands were obtained by densitometry (Labworks Image acquisition, Cambridge, UK) and expressed as a ratio of the housekeeping gene, glyceraldehyde-3-phosphate dehydrogenase (GAPDH-3). Reagents were from Invitrogen, Life Technologies, UK. Adenosine-receptor gene sequences were downloaded from [www.genome.gov/10001772](http://www.genome.gov/10001772). Primers (Table S1) were designed, such that exon-exon boundaries were crossed to ensure they were mRNA specific. Sequences were subjected to the BLAST programme and primers made by Sigma–Genosys Ltd (UK).

**Table S1. Primer sequences used for RT-PCR analysis of adenosine receptors in human detrusor muscle**. Tm, melting temperature.

| Primer | | Direction | Primer sequence | Exon | Base pair number | Tm, °C |
| --- | --- | --- | --- | --- | --- | --- |
| Adenosine receptors | A1 | Sense | 5’-gccacagacctacttccaca-3’ | 5 | 304 | 62.8 |
|  | A1 | Antisense | 5’-ccttctcgaactcacacttg-3’ | 6 | 304 | 65.0 |
|  | A2_A_ | Sense | 5’-aacctgcagaacgtcaccaa-3’ | 1 | 244 | 65.7 |
|  | A2_A_ | Antisense | 5’-gtcaccaagccattgtaccg-3’ | 2 | 244 | 65.2 |
|  | A2_B_ | Sense | 5’-tcctcgagtggtccatcag-3’ | - | 298 | 63.4 |
|  | A2_B_ | Antisense | 5’-tcctcgagtggtccatcag-3’ | - | 298 | 64.1 |
|  | A3 | Sense | 5’-accactcaaagaagaatatg-3’ | 2 | 327 | 53.9 |
|  | A3 | Antisense | 5’-acttagctgtcttgaactcc-3’ | 2 | 327 | 59.9 |
| GAPDH-3 |  | Sense | 5’-gagtcaacggatttggtcgt-3’ | 3 | 103 | 63.9 |
|  |  | Antisense | 5’-ttgaggtcaatgaaggggtc-3’ | 2 | 103 | 63.8 |

*Western blotting.* Total protein lysate (20 µg) was centrifuged (4°C, 1,000g; 15 min) in 4x-NuPAGE LDS and the supernatant used for immunoblotting. Proteins were separated (100 V) on NuPAGE Novex bis-Tris precast gels (Invitrogen, UK), transferred to polyvinylidene difluoride membranes (Amersham Biosciences, UK), washed in PBS and incubated (1 hour, 4°C) in blocking buffer (PBS+0.1% Tween-20 (PBST), 5% powdered skimmed milk). Membranes were probed, 4°C overnight, with rabbit polyclonal antibodies of A-receptors (A1, A2_A_, A2_B_ and A3 - 1:2000 dilution; Alpha Diagnostic Chemicals, UK). After washing in PBST, membranes were incubated (1 hour, 23°C) with goat anti-rabbit IgG-horseradish peroxidase-conjugated secondary antibody (1:5000 dilution; DAKO, Denmark). Antibodies were diluted in blocking buffer. Blots were developed using LumiGLO Reserve Chemiluminescent Substrate and hyperfilm ECLplus (Amersham, UK). Densitometric analyses used Image-J image analysis software (v1.37; rsb.info.nih.gov/ij). Protein band densities were normalised to actin band densities. Gels were run with a biotinylated protein ladder to allow estimation of target bands’ molecular weights (Cell Signaling Technology, Danvers, MA). A positive control used a commercial sample that expresses all four receptor subtypes (ab4008, Abcam Ltd., Cambridge, U.K.) and in each case the molecular weights (mw) corresponded to those in lysates from stable and NDO bladders (A1, A2_B_, A3 mw≈36 kDa; A2_A_ mw ≈ 45 kDa – Yu et al. 2006).

References

Chomczynski P, Sacchi N (1987). Single-step method of RNA isolation by acid guanidinium thiocyanate-phenol-chloroform extraction. Anal Biochem 162: 156-159. PMID: 2440339.

Yu W, Zacharia LC, Jackson EK, Apodaca G. Adenosine receptor expression and function in bladder uroepithelium. Am J Physiol Cell Physiol. 2006; 291: C254-265. PMID: 16571869.
